# Molecular Analysis of Protein-Protein Interactions in the Ethylene Pathway in the Different Ethylene Receptor Subfamilies

**DOI:** 10.3389/fpls.2019.00726

**Published:** 2019-06-07

**Authors:** Mareike Berleth, Niklas Berleth, Alexander Minges, Sebastian Hänsch, Rebecca Corinna Burkart, Björn Stork, Yvonne Stahl, Stefanie Weidtkamp-Peters, Rüdiger Simon, Georg Groth

**Affiliations:** ^1^ Institute of Biochemical Plant Physiology, Heinrich Heine University, Düsseldorf, Germany; ^2^ Institute of Molecular Medicine I, Heinrich Heine University, Düsseldorf, Germany; ^3^ Center for Advanced Imaging, Heinrich Heine University, Düsseldorf, Germany; ^4^ Institute for Developmental Genetics, Heinrich Heine University, Düsseldorf, Germany

**Keywords:** ethylene receptor subfamilies, signaling, protein-protein interaction, microscale thermophoresis, fluorescence lifetime imaging microscopy

## Abstract

Signal perception and transmission of the plant hormone ethylene are mediated by a family of receptor histidine kinases located at the Golgi-ER network. Similar to bacterial and other plant receptor kinases, these receptors work as dimers or higher molecular weight oligomers at the membrane. Sequence analysis and functional studies of different isoforms suggest that the ethylene receptor family is classified into two subfamilies. In *Arabidopsis*, the type-I subfamily has two members (ETR1 and ERS1) and the type-II subfamily has three members (ETR2, ERS2, and EIN4). Whereas subfamily-I of the *Arabidopsis* receptors and their interactions with downstream elements in the ethylene pathway has been extensively studied in the past; related information on subfamily-II is sparse. In order to dissect the role of type-II receptors in the ethylene pathway and to decode processes associated with this receptor subfamily on a quantitative molecular level, we have applied biochemical and spectroscopic studies on purified recombinant receptors and downstream elements of the ethylene pathway. To this end, we have expressed purified ETR2 as a prototype of the type-II subfamily, ETR1 for the type-I subfamily and downstream ethylene pathway proteins CTR1 and EIN2. Functional folding of the purified receptors was demonstrated by CD spectroscopy and autokinase assays. Quantitative analysis of protein-protein interactions (PPIs) by microscale thermophoresis (MST) revealed that ETR2 has similar affinities for CTR1 and EIN2 as previously reported for the subfamily-I prototype ETR1 suggesting similar roles in PPI-mediated signal transfer for both subfamilies. We also used *in planta* fluorescence studies on transiently expressed proteins in *Nicotiana benthamiana* leaf cells to analyze homo- and heteromer formation of receptors. These studies show that type-II receptors as well as the type-I receptors form homo- and heteromeric complexes at these conditions. Notably, type-II receptor homomers and type-II:type-I heteromers are more stable than type-I homomers as indicated by their lower dissociation constants obtained in microscale thermophoresis studies. The enhanced stability of type-II complexes emphasizes the important role of type-II receptors in the ethylene pathway.

## Introduction

The gaseous plant hormone ethylene is decisive for many growth and developmental processes in plants, including fruit ripening, senescence, and the control of biotic and abiotic stress responses, such as pathogen defense and wounding (Kieber and Ecker, 1993; O’Donnell et al., 1996; Penninckx et al., 1998; Bleecker and Kende, 2000). Most of the current knowledge about ethylene biosynthesis and signal transduction has been obtained by genetic, physiological, and biochemical studies in the model plant *Arabidopsis thaliana*. Based on these studies, a family of mainly endoplasmic reticulum (ER)-membrane bound receptors was identified to catalyze the first step in all ethylene-regulated phenomena. These receptors in which their functional state form homo- or heterodimers at the ER membrane act as negative regulators of the ethylene-signaling pathway, following an inverse-agonist model in which ethylene-binding switches off the downstream signal transmission (Bleecker et al., 1988; Hua et al., 1995; Hua and Meyerowitz, 1998; Chen et al., 2002).

Sequence analysis and functional studies disclosed that the receptor family is classified into two subfamilies. In *Arabidopsis*, isoforms ETR1 and ERS1 form subfamily-I, whereas subfamily-II contains receptor isoforms ETR2, ERS2, and EIN4 (Hua et al., 1995, 1998; Sakai et al., 1998). Common to all isoforms is a modular structure known from bacterial sensor histidine kinases. In this case, the main elements are a transmembrane (TM) domain with an ethylene binding site at the amino (N)-terminus facing the ER lumen and a large cytosolic domain comprising of a GAF domain followed by a histidine kinase (HK) domain. Genetic and biochemical studies showed that the GAF domain contributes to the formation of the active dimer and that autophosphorylation activity of the kinase domain is inhibited upon ethylene binding. In addition to the GAF and HK domain receptor isoforms, ETR1, ETR2, and EIN4 carry a response regulator domain (RD) at their carboxy (C)-terminus (Chang et al., 1993; Chen et al., 2002; Voet van Vormizeele and Groth, 2008). Despite their similar overall structure, the individual isoforms contain major differences in these modules. For instance, type-II receptors have an additional fourth transmembrane helix. The function of this additional element is still not clear, although it might function as a targeting signal (Chen et al., 2007). In addition, essential residues for histidine kinase activity are missing in this subfamily. However, *in vitro* Ser/Thr kinase activity was demonstrated for these isoforms albeit autokinase activity seems to play only a minor role in ethylene signaling (Wang et al., 2003; Moussatche and Klee, 2004).

Despite these structural dissimilarities, the general function of the different receptor isoforms and response to the plant hormone is highly redundant. Nonetheless, functional specificity among different isoforms has been described, although the underlying molecular mechanisms are still not fully understood (Binder et al., 2006; Wuriyanghan et al., 2009). But even with the exact signal output of the receptors unknown, previous studies have clearly shown that complex formation of receptors with the Raf-like kinase CONSTITUTIVE TRIPLE RESPONSE 1 (CTR1) and the integral membrane protein ETHYLENE INSENSITIVE 2 (EIN2) is an integral part of the ethylene signaling network in the response to the plant hormone. In this process, CTR1 was shown to phosphorylate EIN2 and that interaction of CTR1 with the receptors is critical for CTR1 kinase activity. The presence of ethylene leads to inactivation of the receptors, thereby inactivating CTR1 in turn resulting in dephosphorylation of EIN2. As a consequence, the C-terminal part of EIN2 (EIN2^479-1294^) is cleaved off by a so far unknown mechanism and translocates to the nucleus. Here, it directly or indirectly stabilizes transcription factors EIN3/EIL1, which activate the transcription of ethylene response genes (Gao et al., 2003; Ju et al., 2012; Qiao et al., 2012; Wen et al., 2012). Remarkably, another mechanism affecting plant ethylene response was shown for the cleaved off EIN2 C-terminus as this digest inhibits the translation of *EBF1/2* mRNA in the cytosol thereby preventing EBF1/2-dependent degradation of the EIN3 master transcription factor (An et al., 2010).

In the past, various approaches analyzing the interaction of type-I receptors and downstream signaling components have been developed. For instance, in our lab, interaction of the type-I receptor ETR1 with EIN2^479-1294^ was demonstrated by *in vivo* FRET-studies and quantified by *in vitro* tryptophan fluorescence quench analysis (Bisson et al., 2009). Moreover, recent studies in our lab highlighted that the conserved nuclear localization signal (NLS) sequence of EIN2 significantly contributes to the EIN2-receptor interaction and in the form of a synthetic octapeptide (NOP1) delays fruit ripening and flower senescence (Bisson et al., 2016; Kessenbrock et al., 2017; Milić et al., 2018; Hoppen et al., 2019). In contrast, related information on ethylene receptor subfamily-II is still sparse.

In the work presented here, we performed quantitative biochemical and spectroscopic studies on purified receptor preparations and downstream elements in order to elucidate the protein-protein interaction (PPI) landscape of both receptor subfamilies. To this end, we established expression and purification for the *Arabidopsis* ETR2 as a prototype of the type-II subfamily. Our studies indicate similar roles in PPI-mediated signal transduction for both receptor subfamilies. To that, we visualized homo- and heteromer formation of type-I and type-II receptors by *in planta* fluorescence lifetime analysis (FRET-FLIM) and anisotropy and quantified these interactions by microscale thermophoresis (MST) on purified recombinant proteins. In the end, our study demonstrates the enhanced stability of type-II receptor complexes compared to complexes consisting of type-I isoforms only stressing an important role of type-II receptors for ethylene signal transduction.

## Materials and Methods

### Production of Recombinant *Arabidopsis* Proteins for *in vitro* Interaction Studies

Codon optimized cDNA encoding full-length AtETR2 (UniProt ID: Q0WPQ2) was purchased from GenScript USA according to the published sequence (NCBI ID: NM_113216.3). The cDNA sequence was flanked by a 5′ *SmaI* recognition site and 3′ *XhoI* recognition site. Expression vector pGEX-4T-1 (GE Healthcare Life Sciences) and synthetic DNA were digested with *SmaI* and *XhoI* and ligated. In the resulting plasmid pGEX-4T-1_AtETR2, the region coding for a thrombin cleavage site was changed to a region coding for a tobacco etch virus (TEV) protease cleavage site (ENLYFQG). To this end, a PCR-based approach with 5′-phosphorylated primers was used (Follo and Isidoro, 2008). Furthermore, a 10 × -His-tag was C-terminally fused to ETR2 to facilitate protein purification. The construct was cloned in two successive PCR reactions (Follo and Isidoro, 2008). The resulting plasmid was verified by sequencing and termed pGEX-4T-1_TEV_ETR2_H_10_. The DNA fragment encoding for the transmembrane domain of ETR1 (aa 1-157) was cloned into expression vector pET16b (Novagen), and base pairs coding for cysteines at positions 4 and 6 in the protein were changed to encode serine. The resulting plasmid was termed ETR1-TM^C4SC6S^. Full-length cDNA sequence encoding for AtCTR1 (UniProt ID: Q05609) was purchased from GenScript USA according to the published sequence (NCBI ID: NM_120454.4). The DNA fragment was cloned into expression vector pET30a (Novagen) by using SLIC, and the coding region for CTR1 was extended by a cyan fluorescent protein termed mCerulean (Li and Elledge, 2007; Rizzo et al., 2007). The resulting plasmid was verified by sequencing and termed pET30a_AtCTR1_mCerulean. Primer sequences used for cloning are listed in Supplementary Table S1.

### Molecular Cloning of AtERS1 and AtERS2 Fusions for *in planta* Interaction Studies

Expression vectors encoding for receptor proteins AtERS1 (UniProt ID: Q38846) and AtERS2 (UniProt ID: P93825) carrying a C-terminal RFP-label were kindly provided by Klaus Harter (Grefen et al., 2008). TOPO-AtERS1 and TOPO-AtERS2 entry clones were prepared by using pENTR Directional TOPO Cloning Kit (Thermo Fisher Scientific) following the manufacturer’s instructions. For transient expression in *Nicotiana benthamiana,* expression vectors encoding ERS1 and ERS2 were prepared *via* Gateway LR-reaction (Thermo Fisher Scientific) using C-terminal mVenus- and mCherry-tagged destination vectors pABindmVenus and pABindmCherry, which are based on vector pMDC7 (Curtis and Grossniklaus, 2003). Expression vector pABindmVmC-ERS2 was cloned using pABindFRET as backbone by Gibson Assembly with amplification of four fragments thereby substituting fluorescent protein (FP) GFP by mVenus (Kremers et al., 2006; Gibson et al., 2009; Bleckmann et al., 2010). The resulting expression vector encodes ERS2 and contains a C-terminal mVenus-mCherry fusion protein. Supplementary Table S1 gives an overview of primers used for cloning.

### Transient Expression of AtERS1 and AtERS2 in *N. benthamiana*

*Agrobacterium tumefaciens* strain GV3101 pMP90 was transformed with expression vectors (pABindmVenus-ERS2, pABindmCherry-ERS2, pABindmCherry-ERS1, pABindmCherry-BTI2, pABindmVmC-ERS2) as well as vector pER-Rb encoding for an ER-marker protein tagged to mCherry (Koncz and Schell, 1986; Nelson et al., 2007). To reduce gene silencing *in planta*, expression vectors were co-transfected with silencing suppressor p19 (Qu and Morris, 2002). Cells were cultured in 2YT medium [1.6% (w/v) peptone, 1% (w/v) yeast extract, and 0.5% (w/v) NaCl], precipitated, and resuspended in AS medium [5% (w/v) sucrose, 5 mM MgSO_4_, 5 mM glucose, 0.01% (v/v) Silwet L77, 450 μM acetosyringone]. *A. tumefaciens* cells containing mVenus- and mCherry-tagged expression vectors were mixed at 1:1 ratio to an optical density at 600 nm (OD_600_) of 0.2 and infiltrated in 4-week-old *N. benthamiana* leaves. Transient gene expression was induced 72 –96 h after infiltration by 20 μM β-estradiol and 0.1% (v/v) Tween 20, and protein expression was analyzed from 16 to 25 h.

### Expression of Recombinant *Arabidopsis* Receptor AtETR2 and Protein Kinase AtCTR1 in *Escherichia coli*

The expression vector encoding for recombinant AtETR2 was transformed into chemically competent *E. coli* C43 (DE3) (Lucigen Corporation) cells. Transformants were precultured in 2YT medium containing 100 μg/ml ampicillin at 30°C for 16 h. Preculture was diluted to an OD_600_ of 0.1 in 500 ml terrific broth (TB) medium (12 g/L peptone, 24 g/L yeast extract, 5 g/L glycerol, 1.8 g/L KH_2_PO_4_, 19.8 g/L K_2_HPO_4_) containing 100 μg/ml ampicillin and 2% ethanol (v/v). Cells were incubated at 30°C with shaking at 180 rpm. At OD_600_ = 0.4 temperature was reduced to 16°C. The bacteria were grown to OD_600_ = 0.6 and heterologous protein expression was induced with 0.5 mM isopropyl β-d-1-thiogalactopyranoside (IPTG). Cells were further grown for 4 h, harvested by centrifugation, flash-frozen in liquid nitrogen, and stored at −20°C. AtCTR1 was expressed in *E. coli* BL21 (DE3) cells. Plasmid pRARE (Novagen) was co-transformed carrying the genes for essential tRNAs encoded by rarely used codons in *E. coli*. Cells were grown at 30°C in 500 ml 2YT medium containing 25 μg/ml kanamycin and 2% ethanol (v/v). Cells were grown to an OD_600_ = 0.4, and temperature was reduced to 16°C. Protein expression was induced at OD_600_ = 0.6 by addition of 0.2 mM IPTG. Cells were further incubated for 20 h at 16°C, harvested by centrifugation, flash-frozen in liquid nitrogen, and stored at −20°C. Protein expression of recombinant proteins was analyzed by SDS-PAGE followed by immunoblotting (Laemmli, 1970; Towbin et al., 1979).

### Purification of Recombinant AtETR2 and AtCTR1

Protein purification steps were performed on ice or at 4°C, if not stated otherwise. Resulting AtETR2 cell pellet was resuspended in PBS lysis buffer [PBS pH 8.0, 10% (w/v) glycerol, 1 mM DTT, 0.002% (w/v) phenylmethylsulfonyl fluoride (PMSF) and 10 mg/L DNaseI (PanReac AppliChem), 5 ml PBS lysis buffer per 1 g cells]. Cells were broken with Constants Cell Disruption System (Constant Systems) at 2.4 kbar and 5°C. Cell lysate was centrifuged for 30 min at 14,000 × *g* to remove cell debris and inclusion bodies. The supernatant was centrifuged again for 30 min at 40,000 × *g*. The resulting membrane pellet was resuspended in PBS lysis buffer and isolation of cell membranes were achieved by centrifugation at 34,000 × *g* for 30 min. Membrane pellets were flash-frozen in liquid nitrogen and stored at −80°C. For protein solubilization, membranes were resuspended in buffer S [50 mM Tris/HCl pH 7.8, 200 mM NaCl, 1.2% (w/v) FosCholine-16, 2.5 mM DTT, 0.002% (w/v) PMSF] and stirred at 700 rpm for 1 h. Membrane fragments were removed by ultracentrifugation at 230,000 × *g* for 30 min. The resulting supernatant was loaded to a 5 ml Ni-NTA HisTrap FF column (GE Healthcare Life Sciences), equilibrated in buffer R [50 mM Tris/HCl pH 7.8, 200 mM NaCl, 2.5 mM DTT, 0.015% (w/v) FosCholine-16]. The column was washed with 10 column volumes (CV) of buffer R, followed by 20 CV buffer R-ATP (buffer R with additional 50 mM KCl, 20 mM MgCl_2_, 10 mM ATP). The column was washed again with 10 CV buffer R, followed by 10 CV buffer R containing 50 mM imidazole. Finally, the receptor AtETR2 was eluted with 250 mM imidazole. To purify His-tagged protein kinase AtCTR1, resulting cell pellet was resuspended in lysis buffer C [50 mM HEPES/NaOH pH 7.6, 300 mM NaCl, 5% (w/v) glycerol, 5 ml lysis buffer C per 1 g cells]. About 10 mg/L DNaseI and 1 × EDTA-free cOmplete Protease Inhibitor Cocktail (Roche) were added to cells prior to cell disruption. Cells were disrupted by passing through a pre-cooled French pressure cell at 12,000 psi (1 psi = 6.9 kPa). The cell lysate was ultracentrifuged at 230,000 × *g* for 60 min. The resulting supernatant was loaded onto a 5 ml Ni-NTA HisTrap HP column (GE Healthcare Life Sciences), equilibrated in buffer C-P [buffer C with 0.002% (w/v) PMSF]. The column was washed with 10 CV of buffer C-P, followed by 20 CV buffer C-ATP [buffer C with additional 50 mM KCl, 20 mM MgCl_2_, 10 mM ATP, 0.002% (w/v) PMSF]. The column was washed with 10 CV buffer C-P containing 50 mM imidazole and 100 mM imidazole, respectively. AtCTR1 was eluted with 500 mM imidazole in buffer C-P. Purified proteins were concentrated in a 50 kDa Amicon Ultra-15 concentrator (EDM Millipore). Buffer was changed by a desalting step on a PD-10 or PD MiniTrap G-25 column (GE Healthcare Life Sciences) depending on subsequent applications.

### Circular Dichroism Spectroscopy of Recombinant *Arabidopsis* Receptor Proteins

**Figure 1:**
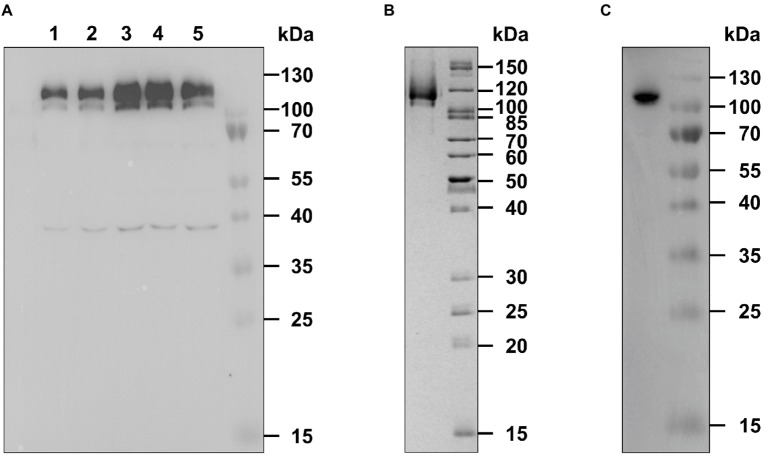
Expression and purification of recombinant AtETR2. **(A)** *E. coli* C43(DE) strain was used for heterologous expression of *Arabidopsis thaliana* receptor ETR2. Expression was analyzed by SDS-PAGE and immunoblotting. Protein expression was monitored 1 (lane 1) to 5 h (lane 5) after induction with IPTG and detected by an anti-His antibody. AtETR2 migrates on SDS gels with an apparent molecular mass of 120 kDa. **(B)** His-tagged AtETR2 was purified by IMAC, separated by SDS-PAGE and visualized by colloidal Coomassie staining and **(C)** immunoblotting using an anti-His antibody.

**Figure 2:**
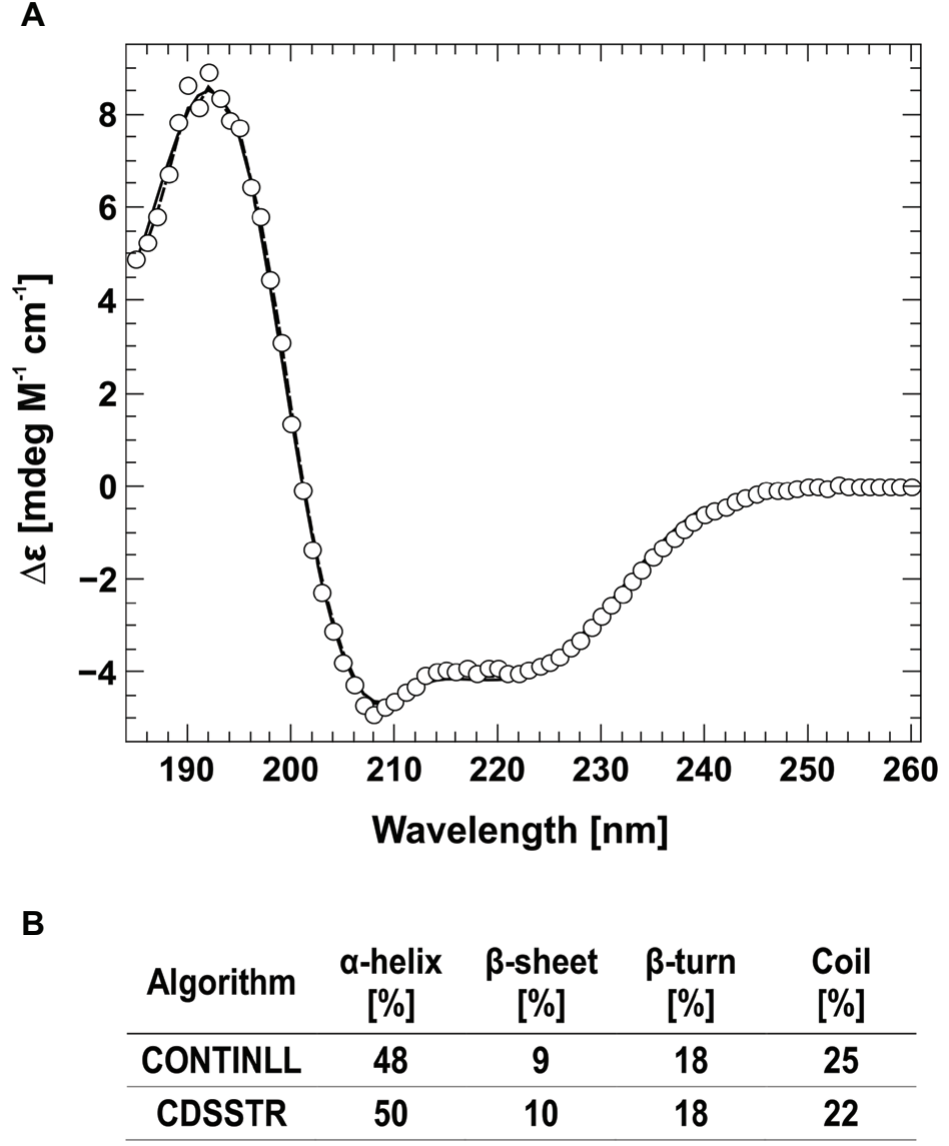
Circular dichroism spectra of AtETR2. **(A)** The far-UV spectra of AtETR2 was calculated and adjusted to molar extinction (∆ɛ) considering molecular weight and protein concentration of AtETR2. **(B)** Secondary structure content was calculated by CONTINLL (solid line) and CDSSTR (dashed line) from the CDpro software package.

AtETR1 and AtETR2 were expressed and purified as described in this article (Figure 1, Supplementary Figure S1A) and in Milić et al., 2018. Purified receptors were characterized by circular dichroism (CD) spectroscopy. For the far-UV spectra, purified *Arabidopsis* receptors were measured at a final concentration of 0.2 mg/ml in CD buffer (10 mM K_2_HPO_4_/KH_2_PO_4_ pH 8.0). Therefore, buffer was exchanged to CD buffer using a PD MiniTrap G-25 column (GE Healthcare Life Sciences) and protein samples were ultracentrifuged at 230,000 × *g* for 30 min. Protein and FosCholine-16 concentrations were determined by a Direct Detect infrared spectrometer (EMD Millipore). In blank samples, FosCholine-16 concentrations were adjusted to correspond to the final detergent concentration in the protein samples. For a detailed protocol for protein preparation, see Kessenbrock and Groth, 2017. CD spectra were recorded by a Jasco J715 spectropolarimeter (Jasco GmbH) using a cylindrical quartz cuvette with 1-mm-path-length (Hellma Analytics). Each protein spectrum was measured from 260 to 180 nm at room temperature and represents an average of 10 continuous scans recorded with a bandwidth of 1 nm at 50 nm/min. Secondary structure content of the purified receptors was calculated using reference protein set SMP50 in programs CDSSTR and CONTINLL from the CDpro software package (see Figure 2, Supplementary Figures S1E,F; Provencher and Glöckner, 1981; Johnson, 1999; Sreerama and Woody, 2000).

### *In vitro* Autokinase Activity Assay of *Arabidopsis* Receptor Proteins

**Figure 3:**
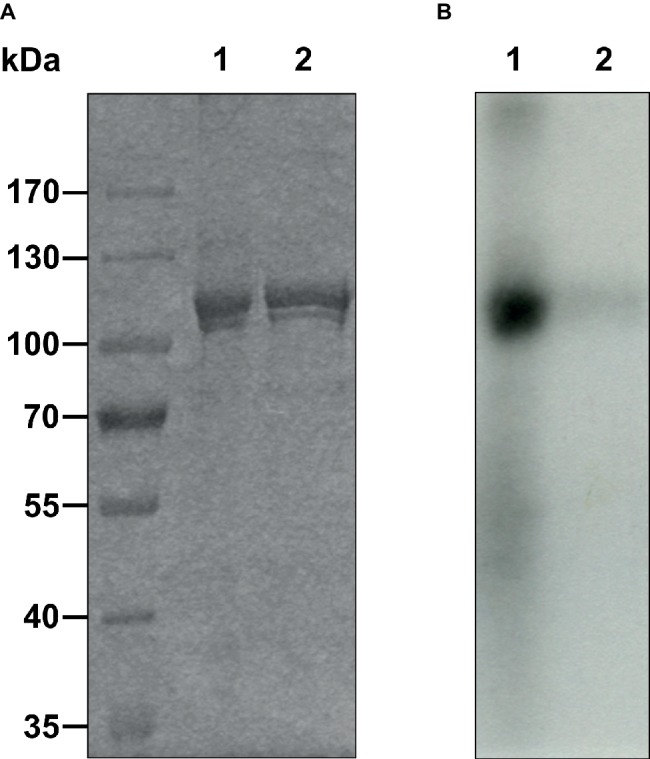
Autophosphorylation of purified AtETR2 was performed with 0.1 mM [γ-^32^P]ATP and magnesium as cofactor. Proteins were detected by **(A)** Coomassie staining. **(B)** Incorporation of ^32^P was measured by autoradiography for 6 days. Experiments were performed using AtETR2 solubilized and purified without ATP purification step (1) or chemically and thermally denatured AtETR2 (2).

Autokinase activity of AtETR1 and AtETR2 was assessed by an *in vitro* kinase assay. To this end, full-length receptors were expressed and purified as previously described either with (Figure 3) or without the additional ATP purification step (see Supplementary Figures S1B–D). Purified proteins (1 mg) were incubated in kinase assay reaction buffer [50 mM Tris/HCl, pH 7.5, 0.1 mM EGTA, 0.1 mM DTT, 10 mM Mg(CH_3_COO)_2_] supplemented with 0.1 mM [γ-^32^P]ATP (Hartmann Analytic) for 30 min at 37°C. Protein denaturation was obtained by the addition of 40 mM DTT and 2% (w/v) SDS for 30 min at 60°C prior to *in vitro* phosphorylation. The kinase reactions were stopped by the addition of SDS sample buffer, and the samples were subjected to SDS-PAGE. After Coomassie staining, the gel was dried and autoradiography was performed for 6 days.

### Fluorescent Labeling for Microscale Thermophoresis Studies

Protein-protein interactions were analyzed by microscale thermophoresis (MST) (Duhr and Braun, 2006; Jerabek-Willemsen et al., 2011). Therefore, recombinant proteins were labeled with Alexa Fluor 488 succinimidyl-ester (Thermo Fisher Scientific). For this purpose, buffer of purified and concentrated AtETR2 and AtCTR1 samples were exchanged on a desalting PD-10 column. Samples were concentrated again resulting in 500-μl protein sample of AtCTR1 in labeling buffer L (50 mM K_2_HPO_4_/KH_2_PO_4_ pH 8.0, 300 mM NaCl) and AtETR2 in buffer L-R [buffer L with 0.015% (w/v) FosCholine-16]. Recombinant AtEIN2^479-1294^ was expressed and purified as previously described (Bisson et al., 2016) and buffer was exchanged to labeling buffer L-E [buffer L with 6% (w/v) glycerol, 10 mM EGTA]. Expression, purification, and labeling of AtETR1 were performed as described in Milić et al., 2018. Alexa Fluor 488 succinimidyl-ester was applied to each protein in 2.5-fold excess and incubated while mixing slightly for 30 min in the dark at ambient temperature. Buffer of labeled proteins was exchanged for AtETR2 to MST buffer 1 [50 mM HEPES/NaOH pH 7.8, 300 mM NaCl, 5% (w/v) glycerol, 0.015% (w/v) FosCholine-16], for AtETR1 to MST buffer 2 [50 mM Tris/HCl pH 7.8, 300 mM NaCl, 5% (w/v) glycerol, 0.015% (w/v) FosCholine-16], and for AtCTR1 and AtEIN2^479-1294^ to MST buffer 3 [50 mM Tris/HCl pH 7.8, 300 mM NaCl, 5% (w/v) glycerol]. The protein samples were centrifuged at 230,000 × *g*, for 30 min, and at 4°C. Protein samples solutions were adjusted to a final glycerol concentration of 20% (w/v), flash-frozen in liquid nitrogen, and stored at −80°C.

### Quantitative Interaction Studies by Microscale Thermophoresis

Protein-protein interactions were analyzed by microscale thermophoresis. Therefore, the receptors AtETR1 and AtETR2 as well as soluble proteins AtEIN2^479-1294^ and AtCTR1 were purified and labeled as previously described. Experiments were performed on a Monolith NT.115 Blue/Green (NanoTemper Technologies) in three independent replicates, whereas for negative controls, measurements were done in duplicates. If not stated otherwise, measurements were performed in standard glass capillaries (NanoTemper Technologies). For binding studies of AtEIN2^479-1294^ (in MST buffer 3) to AtETR2 (in MST buffer 1), proteins were used as follows: 20 nM of labeled AtETR2, 2 μM as the highest, and 0.98 nM as the lowest AtEIN2^479-1294^ concentration. Measurements were performed at 20% MST power. As a negative control, AtEIN2^479-1294^ was heated to 95°C for 5 min, diluted in MST buffer 3 and mixed with 40 mM DTT and 4% (v/v) SDS. Measurements were carried out as described before. Furthermore, interaction of AtETR2 to AtEIN2^479-1294^ was analyzed using 75 nM labeled AtEIN2^479-1294^ and measured with 4 μM as the highest and 1.95 nM as the lowest AtETR2 concentration. Protein samples were incubated at ambient temperature and measured at 60% MST power. For quantification of receptor AtETR2 and AtETR1 binding to CTR1 (in MST buffer 3), receptors AtETR2 and AtETR1 (in MST buffer 2) were measured with 4 μM as highest and 0.98 nM as lowest receptor concentration. AtETR1 was mixed in a 1:1 volume ratio with labeled AtCTR1 (50 nM final concentration) and measured at 60% MST power. For AtETR2 binding to AtCTR1, labeled AtCTR1 was used in a final concentration of 20 nM. The protein mixture was incubated for 10 min at ambient temperature, transferred into premium glass capillaries, and measured at 60% MST power. As a negative control, the protein mixture was incubated directly after the 10 min incubation step with 4% (v/v) SDS and 80 mM DTT for 5 min in the dark at RT, resulting in the same protein concentrations as before mentioned. For quantification of receptor-receptor interactions, labeled receptors were used at a final concentration of 40 nM. Labeled receptors were mixed in a 1:1 ratio with non-labeled AtETR1, AtETR2 (4 μM as the highest and 0.49 nM as the lowest receptor concentration) or ETR1-TM^C4SC6S^ (MST buffer 2, 16 μM as the highest and 0.98 nM as the lowest concentration), thereby adjusting the detergent concentration to 0.0075% (w/v) FosCholine-16. Sample mixtures containing AtETR2 (labeled as well as non-labeled) were initially incubated for 10 min at RT and transferred into premium glass capillaries. All receptor-receptor binding studies were performed at 60% MST power. As negative controls, denaturation buffer (4% (v/v) SDS, 40 mM DTT in MST buffer 1 or 2, respectively) was added to the receptors. Samples were incubated for 5 min at RT in the dark and MST measurements were carried out as described for the native receptor proteins. All dissociation constants (*K*_d_) were calculated to a binding model assuming a 1:1 stoichiometry per binding partner.

### Confocal Fluorescence Microscopy

*N. benthamiana* epidermis cells were imaged for protein expression and protein localization using a LSM 780 laser-scanning confocal microscope (Carl Zeiss GmbH) using a C-Apochromat 40×/1.2 W Corr M27 objective with a zoom factor of 4. The pinhole was set to 1 Airy Unit (AU). The following settings were used: 488/561 beam splitter with 488 nm excitation for mVenus and 561 nm excitation for mCherry. Fluorescence was detected between 508–552 nm for mVenus and 570–624 nm for mCherry by a GaAsP detector. In combination with FM4-64 the detection wavelength of mVenus was between 490 and 552 nm. FM4-64 was detected between 562 and 626 nm. The laser strength was adjusted between 2 and 9% for mVenus, with a gain of 700–900, whereas for mCherry, a laser strength between 1 and 6% with a gain of 750–980 was used. Staining of plasma membrane was carried out using 10 μM FM4-64 which was infiltrated 20 min before image acquisition. Images were recorded and processed using Fiji software (Schindelin et al., 2012).

### Fluorescence Lifetime Imaging Microscopy in *N. benthamiana* Leaves

Fluorescence lifetime imaging microscopy (FLIM) was performed using a LSM 780 confocal laser-scanning microscope additionally equipped with a single-photon counting device enabling picosecond time resolution (PicoQuant Hydra Harp 400). mVenus fluorescence was excited at 485 nm with a rate of 32 MHz with a linearly polarized pulsed diode laser (LDH-D-C-485, PicoQuant). The pinhole was set to 1 Airy Unit (AU). Excitation power was adjusted to 1 μW at the objective C-Apochromat (40×/1.2 W Corr M27) prior to measurements. mCherry was excited at 561 nm by a continuous wave laser with a laser strength of 0.1%. Emitted light of mVenus was separated into its parallel and perpendicular polarization. mVenus fluorescence was detected by Tau-SPADs (PicoQuant) in a narrow range of its emission spectrum (band-pass filter: 534/30, AHF). mCherry fluorescence detection was set by the band-pass filter (HC 607/70, AHF). Images were acquired with 256 × 256 pixel, zoom factor 4, 12.61 μs pixel dwell time, and a resolution of 210 nm/pixel. A series of 80 frames were merged into one image and further analyzed using SymphoTime64 (PicoQuant).

### Fluorescence Lifetime and Anisotropy Analysis

The fluorescence lifetime of mVenus was analyzed using the software tool SymPhoTime 64, version 2.3 (PicoQuant, Berlin, Germany). Due to low excitation power to prevent photobleaching during image acquisition and the small pixel size to gain spatial resolution, the number of photons per pixel was still low after merging of frames. An individual ROI for every dataset was generated ensuring that only pixels with a minimum number of 100 photons contributed to the decay histogram. In the donor-only case, a mono-exponential fit model, including background contribution and shifting of the instrument response function, was sufficient to describe the decay histogram of mVenus fluorescence and extract the fluorescence lifetime. In the case of FRET an additional exponent was used to describe the decay and a mean fluorescence lifetime was extracted from the resulting fit.

The steady-state anisotropy r is given by $r=\frac{I_{par}-\;GI_{\mathrm{per}}}{I_{par}+\;2GI_{\mathrm{per}}}$. *I_par_* and *I_per_* are the average fluorescence count rates per pixel with the emission parallel (*I_par_*) and perpendicular (*I_per_*) to the excitation polarization direction (Gauthier et al., 2001; Sarkar et al., 2009). Orientation sensitivity differences of the detection system were corrected by determining the *G*-factor by calibration measurements using Rhodamine110. Data were statistically evaluated with GraphPad Prism using Student’s *t*-test with Welch correction and Mann-Whitney test, respectively.

## Results

### Heterologous Expression and Purification of *A. thaliana* Type-II Receptor ETR2

Previous studies have revealed the direct interaction of *A. thaliana* receptor ETR1 (AtETR1) with the C-terminus of EIN2 (aa 479-1294) *in planta.* These *in vivo* results were supported by tryptophan fluorescence quench studies on purified proteins which disclosed that receptors interact with the EIN2 downstream ethylene signaling protein with high affinity. Along the same lines, *in planta* FRET studies have demonstrated the interaction of type-II receptors and EIN2 (Bisson et al., 2009; Bisson and Groth, 2010). However, these studies did not reveal binding mode and binding affinities of the EIN2 C-terminus with type-II receptors. To clarify whether type-II and type-I receptors interact with EIN2 in a similar way and affinity as the type-I receptor subfamily, we heterologously expressed and purified ethylene receptor ETR2 from *A. thaliana* (AtETR2) as prototype of the type-II subfamily. To this end, AtETR2 expression plasmid pGEX-4T-1_TEV_ETR2_H_10_ was transformed into *E. coli* strain C43 (DE3) which has been successfully used for expression of AtETR1 (Voet van Vormizeele and Groth, 2008). Bacterial cells harboring the AtETR2 expression plasmid show an increased expression of a protein band with an apparent molecular weight of 120 kDa up to 4 h after induction, whereas 5 h after induction the corresponding protein band appear faded suggesting degradation of the over-expressed receptor by bacterial proteases (Figure 1A, line 5). On the basis of the expression studies showing maximum protein production 4 h after induction, cells were harvested 4 h post-induction and solubilized from the host membranes by the detergent FosCholine-16. Solubilized AtETR2 receptors were purified by metal-chelate affinity chromatography on Ni-NTA agarose (GE Healthcare). High purity of the resulting receptor preparations is indicated by the single band observed on the SDS protein gel (Figure 1B). Identity of the purified protein band with AtETR2 was confirmed by immunoblotting with an anti-His Tag antibody (Figure 1C).

### Analysis of Secondary Structure and Functional Folding of AtETR2

The folding and secondary structure of AtETR2 was analyzed by circular dichroism (CD) spectroscopy. The CD spectra has two minima at 208 and 221 nm and an isosbestic point at 201 nm (Figure 2), indicating a predominately α-helical structure of the purified AtETR2. Secondary structure content was quantified by CONTINLL and CDSSTR—two different algorithms for secondary structure assignment—at 48–50% α-helix and 9–10% β–sheet demonstrating that the recombinant protein adopts a well-folded structure and is likely to reflect a native confirmation of the *Arabidopsis* type-II ethylene receptor ETR2. Additionally, AtETR2 functionality was probed by an *in vitro* radiolabeling autokinase assay. For that, AtETR2 was purified without ATP pre-incubation. Purified protein preparations were then incubated with [γ-^32^P]ATP. Autoradiography of the SDS gel loaded with samples from the kinase assay revealed incorporation of ^32^P for those samples incubated in the presence of magnesium—an essential cofactor for histidine kinase activity—but not for those containing chemically denatured AtETR2 (Figure 3). These results further confirm functional folding of purified AtETR2. Notably, the purified type-II receptor shows somewhat reduced kinase activity in the presence of manganese (Supplementary Figure S1D), which is in line with results of previous studies using the ETR2∆GAF mutant (Moussatche and Klee, 2004). To allow for comparison to the type-I receptor subfamily in terms of protein affinities and protein activities, similar buffer and detergent conditions were applied for purification of AtETR1. The corresponding autokinase assay (Supplementary Figure S1B) supports that both receptor subfamilies show similar phosphorylation activities at these conditions and once again emphasizes the purification of the full-length receptors in a functional, catalytically active state.

### Microscale Interaction Studies of AtETR2 and Downstream Signaling Components AtEIN2^479-1294^ and CTR1

**Figure 4:**
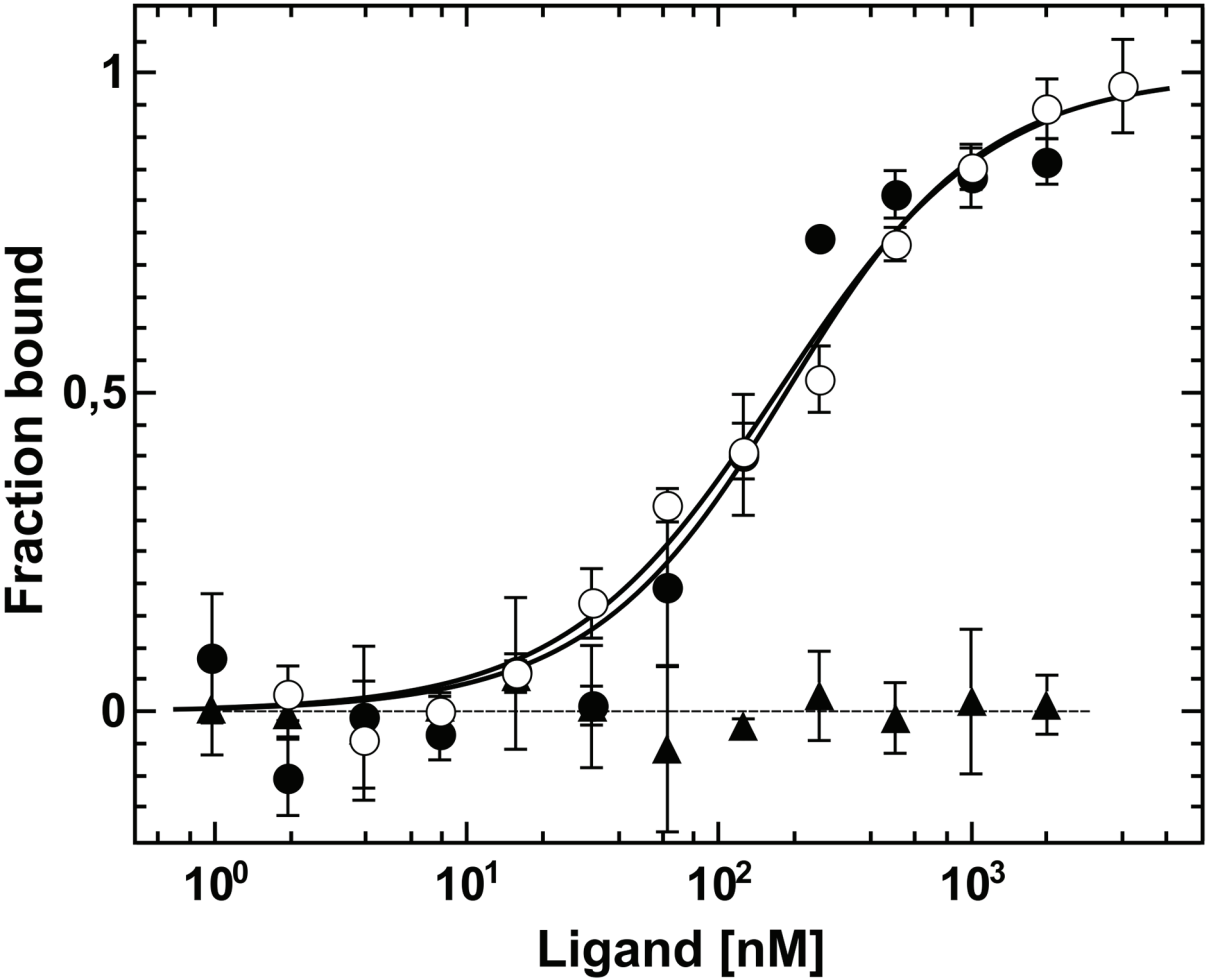
Interaction studies of *Arabidopsis* ETR2 and EIN2 by MST. Dissociation constants of the interactions were obtained from the related binding curves. Titration of unlabeled AtEIN2^479-1294^ to AtETR2 (●) is described by a dissociation constant (*K*_d_) of 161(30) nM. Chemically and thermally denatured AtEIN2^479-1294^ shows no binding event to AtETR2 (▲). Binding of unlabeled AtETR2 to AtEIN2^479-129^ is represented by a *K*_d_ value of 147(15) nM (○). All data represent the mean (SD) of three independent measurements (●, ○) and duplicates (▲), respectively.

**Figure 5:**
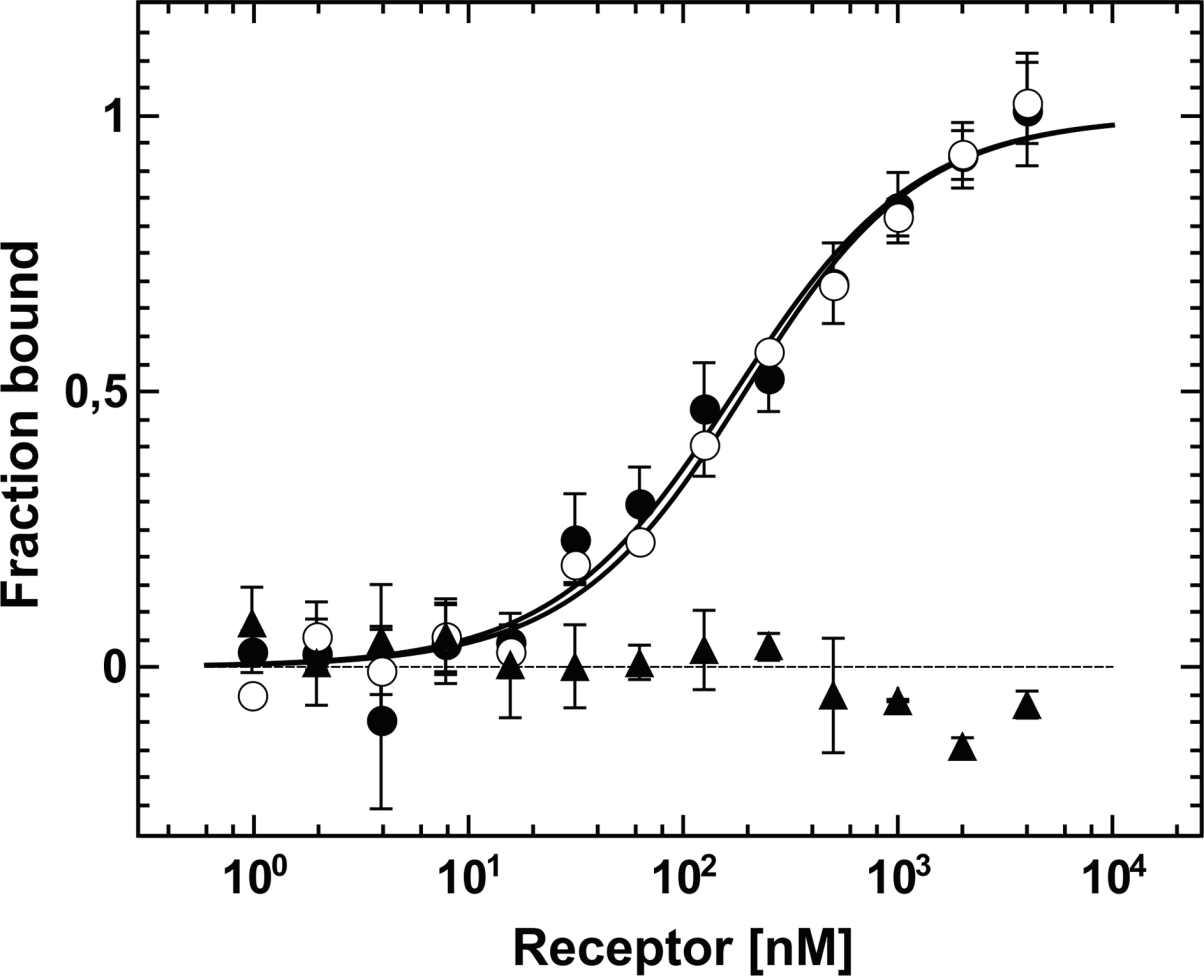
MST based protein-protein interaction assay between AtCTR1 and receptor proteins AtETR1 and AtETR2. Binding of AtETR1 to fluorescently labeled AtCTR1 measured by MST resulted in a *K*_d_ value of 169(15) nM (○). For AtCTR1-AtETR2 complex formation a *K*_d_ value of 165(20) nM was obtained (●). As negative control, titration of chemically denatured AtCTR1 with AtETR2 is shown. Here, no binding event was observed (▲). Data are given as the mean (SD) of independent triplicates (●, ○) and duplicates (▲), respectively.

Protein-protein interactions of purified receptors with downstream ethylene signaling components EIN2 and CTR1 were monitored and quantified by microscale thermophoresis (MST). This biophysical technique is based on the motion of molecules in a temperature gradient and strongly depends on the charge, hydration-shell and size of the moving molecules. At least one of these qualities typically changes upon complex formation. Hence, thermophoresis provides a sensitive and reliable method to analyze and to quantify protein-protein interactions (Duhr and Braun, 2006; Wienken et al., 2010; Jerabek-Willemsen et al., 2011). For a start, we applied this technique to quantify the interaction of the soluble EIN2 C-terminus (AtEIN2^479-1294^) with the type-II receptor prototype AtETR2. Both recombinant proteins were purified as previously described (see Figure 1A, Supplementary Figure S2B; Bisson et al., 2016). Addition of EIN2^479-1294^ to labeled AtETR2 shows clear changes of thermophoresis and thermophoretic signals obtained with increasing EIN2^479-1294^ concentrations follow a clear binding curve. From this binding curve, an apparent dissociation constant (*K*_d_) of 161(30) nM was obtained which is indicative of a tight and highly specific interaction of AtEIN2^479-1294^ with the AtETR2 receptor. Similarly, clear changes of the thermophoretic signal were observed upon addition of AtETR2 to labeled EIN2^479-1294^. The corresponding binding curve shows an apparent *K*_d_ of 147(15) nM. Any potential effect of the fluorescence probe Alexa Fluor 488 which is positioned at different sites in the two complementary titration set-ups on the interaction, integrity or stability of any of the two binding partner can be ruled out as both binding studies show almost the same low nanomolar *K*_d_ value (Figure 4). The tight and highly specific interaction of AtETR2-AtEIN2^479-1294^ is further supported in titration studies using denatured AtEIN2^479-1294^ as negative control. Here, no interaction between both binding partners was detectable. Like EIN2 the Raf-like protein kinase CTR1 is another signaling element downstream of the *Arabidopsis* ethylene receptor family. Recombinant AtCTR1 required for *in vitro* binding studies with receptors AtETR1 and AtETR2 respectively, was purified according to the methods section (see Supplementary Figure S2A). As described before for EIN2, protein-protein interactions of receptor proteins and AtCTR1 were analyzed by MST. To this end, AtCTR1 was labeled with the fluorescent dye Alexa 488 and was mixed with increasing concentrations of AtETR1 or AtETR2 until saturation. Binding of receptors to AtCTR1 detected as changes in thermophoresis correspond to dissociation constants (*K*_d_) of 169(15) nM for the AtCTR1-AtETR1 interaction and of 165(20) nM for the AtCTR1-AtETR2 complex. Again, these numbers indicate a tight and highly specific interaction. Furthermore, it should be noted that both receptor subfamilies show similar affinities for AtCTR1 (Figure 5).

### *In vitro* Quantification of Type-I-Type-II Receptor Interactions

**Figure 6:**
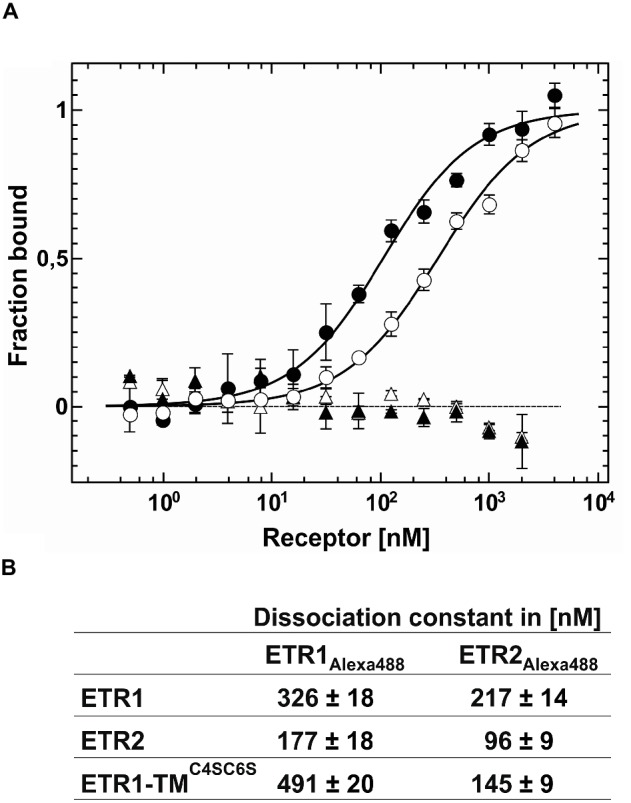
Quantification of receptor-receptor interactions by microscale thermophoresis. **(A)** For the homomeric AtETR1-AtETR1 complex formation a *K*_d_ value of 326(18) nM (○) was obtained. As negative control chemically denatured AtETR1 was used showing no binding event (△). From the binding curve of the homomeric AtETR2-AtETR2 complex a *K*_d_ value of 96(18) nM (●) was calculated. Chemically denatured AtETR2 indicates no interaction of the binding partners (▲). All data represent the mean (SD) of independent triplicates (○, ●) and duplicates (△, ▲). **(B)** Summary of the dissociation constants *K*_d_ for receptor-receptor interactions obtained by MST, also see Supplementary Figure S3. All data represent the mean (SD) of three independent measurements.

To analyze the mode of receptor-receptor interactions and to quantify binding affinities, we studied protein-protein interactions within receptor subfamilies by microscale thermophoresis. To this end, we analyzed homomeric and heteromeric receptor complexes of AtETR1 and AtETR2. For that, either AtETR1 or AtETR2 was labeled with Alexa Fluor 488, and thermophoresis was recorded after the addition of the corresponding binding partner. Binding affinity and dissociation constants indicate strong binding for both receptor subfamily homomers, AtETR1-AtETR1 and AtETR2-AtETR2 (Figure 6A). However, a three-fold higher affinity of the type-II AtETR2-AtETR2 homomer [*K*_d_ of 96(9) nM] was detected in comparison to the type-I AtETR1-AtETR1 homomer [*K*_d_ of 326(18) nM]. In addition, our binding studies revealed that type-I and type-II receptors can also form tight heteromeric complexes with binding constants of 177(18) nM (AtETR1-AtETR2) and 217(14) nM (AtETR2-AtETR1), respectively (Figure 6B, Supplementary Figures S3A,B). Notably, type-II:type-I heteromeric complexes seem to be more stable than type-I homomers. No interactions were detected in the related binding studies when chemically denatured receptors were used (Figure 6A). Previous studies suggested that dimer and higher complex formations in the ethylene receptor family are substantially mediated by their GAF-domains (Grefen et al., 2008; Mayerhofer et al., 2015). However, the role of the receptor transmembrane-domain in complex formation was not resolved in these studies. To address this issue, we analyzed the interaction between the isolated AtETR1 transmembrane domain (ETR1-TM) and full-length receptors AtETR1 and AtETR2 *via* MST. To eliminate any stabilizing effect by disulfide bond formation (Schaller et al., 1995), cysteines in ETR1-TM were substituted to serines (C4SC6S). In the related titration experiments, tight binding of ETR1-TM^C4SC6S^ to both receptor subfamily representatives (AtETR1 and AtETR2) was observed. In line with previously observed heteromeric interactions of full-length receptors, the affinity for AtETR2 was higher than for AtETR1 (Figure 6B, Supplementary Figures S3C,D). Taken together, the binding studies with the isolated transmembrane domain (AtETR1-TM^C4SC6S^) as well as with full-length receptors emphasize that both receptor subtypes interact in homomeric and heteromeric complexes in a selective and specific manner. Moreover, the higher stability of type-II receptor homo- and heteromers highlights the importance of this subfamily to form receptor dimers or higher order oligomers in ethylene signaling.

### *In planta* Detection of Receptor-Receptor Interactions *via* FRET-FLIM Microscopy

**Figure 7:**
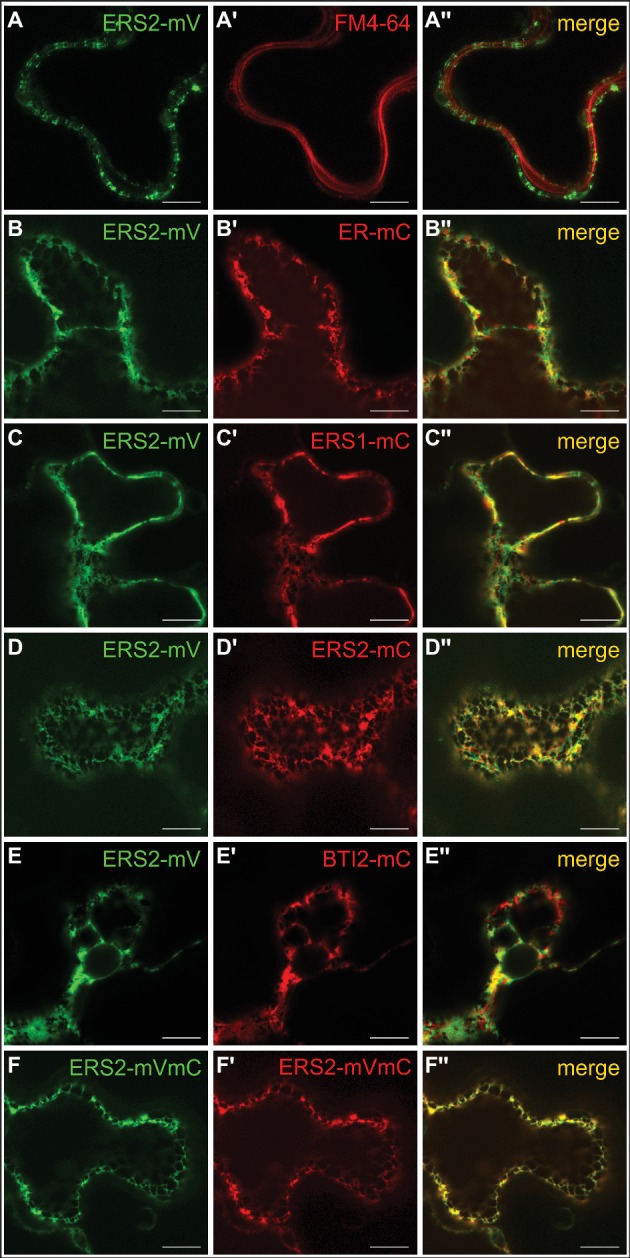
Intracellular localization of AtERS1 and AtERS2 transiently expressed in *N. benthamiana* epidermis cells. Confocal laser scanning microscopy images of mVenus (mV) and mCherry (mC)-tagged receptor proteins. **(A–A″)** AtERS2 does not colocalize with the PM dye FM4-64 and is instead found **(B–B″)** at the ER, where colocalization with the ER-mCherry marker protein is detected. AtERS2-mVenus colocalizes with **(C–C″)** AtERS1-mCherry, **(D–D″)** AtERS2-mCherry and **(E–E″)** BTI2-mCherry at the ER. **(F–F″)** AtERS2 tagged to mVenus and mCherry is also detected at the ER. Bars = 10 μm.

**Figure 8:**
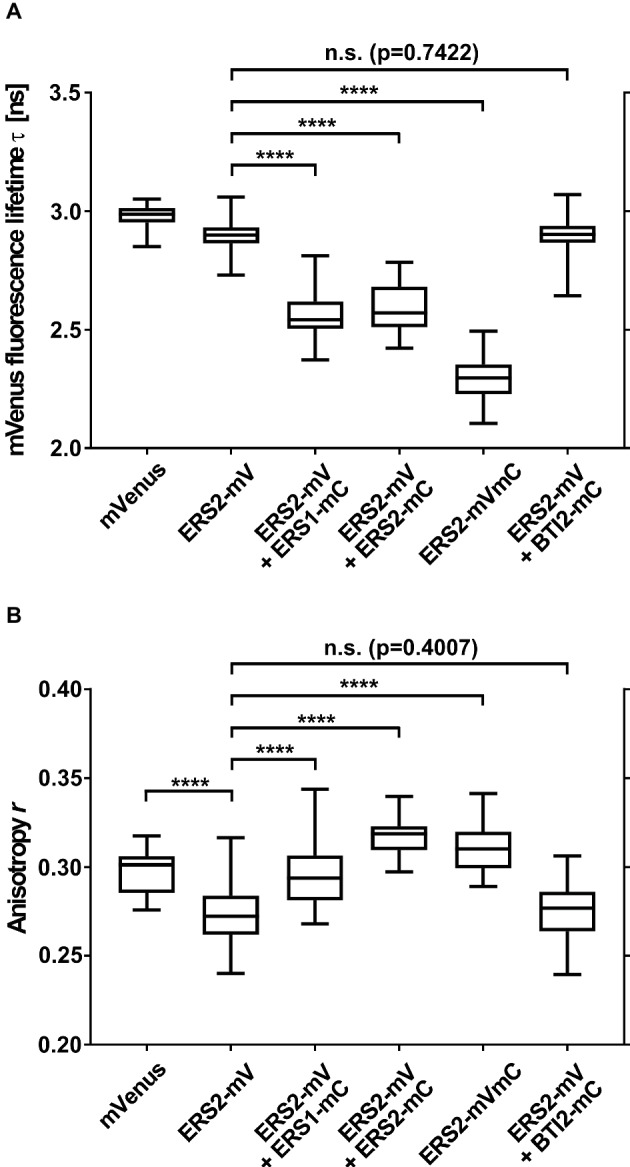
Homo- and heteromeric interaction pattern of ethylene receptors. Depicted are **(A)** fluorescence lifetime (*τ*) and **(B)** anisotropy (*r*) of AtERS2-mVenus coexpressed with mCherry-tagged receptor proteins in *N. benthamiana* leaf epidermal cells. *N. benthamiana* leaf cells were transfected with the indicated proteins, protein expression was induced by β-estradiol and samples were analyzed by confocal microscopy within 16–25 h. Fluorescence lifetime and anisotropy were calculated of free fluorophore mVenus (*n* = 34), donor-only AtERS2-mVenus (*n* = 113) and coexpression of AtERS2-mVenus with either AtERS1-mCherry (*n* = 68) or AtERS2-mCherry (*n* = 18). Coexpression of AtERS2-mVenus with BTI2-mCherry (*n* = 62) was used as negative control, whereas expression of AtERS2-mVenus-mCherry (*n* = 50) was used as positive control. Distribution of mVenus fluorescence lifetime (*τ*) and anisotropy (*r*) are depicted as box plot: median, first and third quartiles, minimum and maximum, error bars indicate minimum and maximum of distribution. Statistical analysis was performed using Mann-Whitney test (**A**, AtERS2-mVenus + AtBTI2-mCherry) and Welch’s *t*-test (all other), ****p < 0.0001, mV = mVenus, mC = mCherry. See also Supplementary Tables S2, S3 for data.

To analyze ethylene type-II:type-I receptor complex formations *in vivo*, we performed fluorescence lifetime imaging microscopy (FLIM). FLIM is used in plant cells to study molecular interactions and detects fluorescence resonance energy transfer (FRET) between two fluorescent-tagged proteins in close proximity (Gadella et al., 1993; Valeur, 2001; Stahl et al., 2013; Long et al., 2017). By further analysis of the donor anisotropy, this method allows the discrimination between hetero-FRET (FRET between two different donor and acceptor fluorophores) and homo-FRET (energy migration between identical fluorophores and thus identical receptors). Therefore, monomeric versions of the fluorescent proteins Venus and Cherry fused to the C-terminus of the *Arabidopsis* ethylene receptors were chosen as FRET pair (Shaner et al., 2004; Kremers et al., 2006). Tobacco epidermal leaf cells were (co-)transformed with the relevant receptor(s), and expression was induced by the addition of β-estradiol. The fluorescence lifetime measurements were performed in combination with an inducible expression system which allows the discrimination of autofluorescence and prevents overexpression artifacts (Suhling et al., 2015). To verify localization at the ER membrane in the tobacco leaf cells, we probed the intracellular localization of full-length type-II receptor AtERS2 by confocal microscopy. As a result, we observed a strictly separated localization of the PM-localized dye FM4-64 and AtERS2-mVenus, whereas a colocalization of AtERS2-mVenus with a mCherry-labeled ER-marker protein was detected (Figures 7A,B; Nelson et al., 2007). Additionally, we detected colocalization of AtERS2 and other *Arabidopsis* ethylene receptors which are a necessary condition for subsequent analysis of receptor-receptor interactions (Figures 7C–F). Type-I and type-II receptors AtERS1 and AtERS2, the ER-bound receptor Reticulon-like protein B2 (AtBTI2) as a negative control, and a tandem construct with both FPs fused to AtERS2 as a positive control were used to pinpoint *in vivo* interaction of the two receptor subfamilies. For each receptor combination, fluorescence lifetime (*τ*) and anisotropy (*r*) of the donor fluorophore mVenus were measured. Fluorescence lifetime of a fluorophore represents the average time a fluorophore remains in the fluorescent state after excitation by a pulsed laser. For heteromeric interactions, the mVenus fluorescence is quenched by FRET between mVenus and mCherry fluorophores, thereby shortening the mVenus fluorescence lifetime (Gadella et al., 1993, 1994), whereas fluorescence anisotropy represents the rotational freedom of a fluorophore. This rotation is reduced by fusion of the fluorophore to a protein, increasing the anisotropy value *r*. Occurrence of hetero-FRET additionally increases the anisotropy due to a direct interaction of donor and acceptor fluorophore, again limiting rotation of the donor fluorophore. Whereas in case of homo-FRET, energy can migrate radiation free from one excitated mVenus fluorophore to another mVenus in close proximity but with slightly different dipole orientation. This leads to a reduced overall mVenus anisotropy due to a depolarization of the overall mVenus signal and thus decreases the observed value *r* for the steady state anisotropy. Thus, the measurement of anisotropy enables a direct discrimination between homo- and heteromeric interactions (Gauthier et al., 2001; Bader et al., 2011). In our studies, we measured lifetimes for each AtERS2-mVenus combination by FLIM. As shown in Figure 8A cotransfections of AtERS2 with either type-I receptor AtERS1 (2.56 ns) or type-II receptor AtERS2 (2.59 ns) resulted in a significant reduction of the AtERS2-mVenus (2.90 ns) fluorescence lifetime (see Supplementary Table S2). In the context of the also observed lifetime reduction of the positive control (AtERS2-mVenus-mCherry) and the unchanged lifetime of the negative control (AtERS2-mVenus:AtBTI2-mCherry), these data indicate heteromeric interactions of the type-I receptor AtERS1 with type-II AtERS2 as well as homomeric interactions of AtERS2 protomers. Formation of AtERS2 homomers is further supported by the observed changes in fluorophore anisotropy (see Figure 8B). In our experiments, we observed a decreased anisotropy (*r =* 0.27) for AtERS2-mVenus compared to free mVenus (*r* = 0.30, see Supplementary Table S3). Moreover, formation of type-II homomers can also be inferred from measurements of AtERS2 with AtERS1. Here, the anisotropy of AtERS2 (*r* = 0.29) is increased compared to the mVenus donor only sample, but decreased compared to the free mVenus fluorophore. In accordance to these results, no significant changes were observed for the negative control compared to the AtERS2-mVenus donor. These data are indicative for homo-FRET interactions between AtERS2-mVenus itself only. In summary, our *in planta* fluorescence studies demonstrate homomeric as well as heteromeric interactions of the type-II receptor AtERS2 *in vivo*. Furthermore, we were able to show that at these conditions homo-FRET interactions of AtERS2 take place as well in the presence as in the absence of type-I receptor AtERS1.

## Discussion

Previous studies on *Arabidopsis* revealed that the ethylene signal perception and transduction is mediated by five receptors localized at the ER membrane (Hua and Meyerowitz, 1998; Grefen et al., 2008). Furthermore, genetic studies identified CTR1 and EIN2 as critical regulators mediating ethylene signaling and identified both proteins as direct interaction partner of the ethylene receptor family (Kieber et al., 1993; Alonso et al., 1999; Huang et al., 2003). However, previous protein-protein interaction studies mainly focused on the prototype type-I receptor AtETR1, whereas related information on subfamily-II receptors is still sparse. Although a direct interaction of ethylene type-II receptors with either CTR1 or EIN2 has been demonstrated by yeast two-hybrid assays and *in planta* FRET studies (Cancel and Larsen, 2002; Bisson and Groth, 2010), detailed information about these interactions is still missing. To elucidate type-II receptor interactions with their downstream signaling targets in more detail, we have established a purification protocol for the *Arabidopsis* full-length type-II receptor ETR2. The protocol is based on the expression of AtETR2 in *E. coli* strain C43 (DE3), which has been successfully applied for the expression of type-I receptor AtETR1 and several other membrane proteins in the past (Miroux and Walker, 1996; Voet van Vormizeele and Groth, 2008). In this case, solubilization of AtETR2 was obtained by the zwitterionic detergent FosCholine-16 and a purification protocol similar to AtETR1 (Classen and Groth, 2012; Milić et al., 2018) was applied. Functional folding of the purified recombinant AtETR2 was verified by CD spectroscopy (see Figure 2). Secondary structure calculations based on CD measurements on the purified receptor determined an α–helical content of 48–50% and a β–sheet content of 9–10% which correspond well to sequence based secondary structure predictions by SOPMA (α–helical: 48% and a β–sheet: 14%) (Geourjon and Deléage, 1995; Sreerama and Woody, 2000; Kessenbrock and Groth, 2017).

Previous studies revealed that the ethylene receptors function as negative regulators and are in their active state in the absence of ethylene (Hua and Meyerowitz, 1998). *In vitro* phosphorylation assays demonstrated that receptors in their active state show autokinase activity of various degrees depending on the divalent cation used. Along these lines, our studies on purified full-length AtETR2 substantiate that the recombinant type-II receptor has higher autokinase activity in the presence of magnesium than in the presence of manganese, which is in accordance with previous studies on truncated AtETR2 lacking the transmembrane domain (Moussatche and Klee, 2004). On the other hand, almost no measurable phosphorylation activity was found for chemically denatured AtETR2 (see Figure 3, Supplementary Figures S1C,D). Taken together, our structural and functional studies on the purified AtETR2 attest that the recombinant type-II receptor used in our *in vitro* binding studies was isolated in a functional and active state. Stable complex formation of type-II receptor AtETR2 with AtEIN2 has been previously identified by *in vivo* FRET studies. Nevertheless only the AtETR1-AtEIN2 interaction was further characterized in detail by *in vitro* tryptophan quenching studies due to the lack of a purified functional type-II receptor isoform at that time (Bisson et al., 2009; Bisson and Groth, 2010). Here, we unravel a tight and highly specific complex formation of AtETR2 with the soluble cytosolic domain of EIN2 (AtEIN2^479-1294^, see Figure 4) which reinforces the previously mentioned *in planta* FRET studies (Bisson and Groth, 2010). Noteworthy, our studies disclose similar binding affinities and binding modes for AtETR1 and AtETR2 with AtEIN2^479-1294^ which substantiate similar roles of the different isoforms in PPI-based signal transfer to their downstream target. Still, the observed similar complex stability is quite surprising considering the low sequence identity of only 39% of both isoforms as well as the pronounced differences of both receptor subfamilies in their kinase activity and transmembrane architecture. However, these differences in the receptor isoforms may only come into effect at specific signaling conditions as previously shown for AtETR1 where receptor phosphorylation or binding of an ethylene agonist were shown to modulate complex stability with EIN2 (Bisson and Groth, 2010).

Genetic and biochemical studies indicate that apart from EIN2 the Raf-like kinase CTR1 also interacts with all members of the ethylene receptor family to mediate ethylene signaling at the ER membrane (Cancel and Larsen, 2002; Gao et al., 2003). Consequently, we also analyzed the interaction of the different receptor subfamilies with CTR1 in this study. As for EIN2, we found similar affinities of the CTR1 kinase with full-length receptors AtETR1 and AtETR2 representing prototypes of both subfamilies. These results differ from previous studies using yeast two-hybrid screening and pull-down assays which propose a preferred interaction of AtCTR1 with AtETR1 (Clark et al., 1998; Cancel and Larsen, 2002), although these studies did not provide clear quantitative analysis. Moreover, pull-down assays were performed with truncated receptor constructs which also affect complex stability with the CTR1 kinase. Taken together, our binding studies determine that both receptor subtypes have similar binding affinities and binding modes with their downstream targets AtEIN2^479-1294^ and AtCTR1 emphasizing similar roles of both receptor subfamilies in PPI-based signal transduction. Bearing in mind that both, CTR1 and EIN2, have been localized to the ER these results correlate well with the idea of ER-borne signaling complexes consisting of various combinations of receptor subtypes, EIN2 and CTR1 (Gao et al., 2003; Bisson et al., 2009; Bisson and Groth, 2010).

For further analysis of receptor complex formation and the role for ethylene signaling *in planta*, we used fluorescence microscopy of transiently expressed receptor proteins in *N. benthamiana* leaf cells. In their native background *Arabidopsis* receptors are localized at the ER membrane. In order to demonstrate that this localization is not affected at overexpressed conditions in the *N. benthamiana* system, we first determined subcellular localization of transiently expressed full-length type-II receptor AtERS2 by confocal microscopy. In our experiments, AtERS2 shows clear separation from the plasma membrane as evidenced by staining with FM4-64. On the other hand, clear colocalization with an ER-marker protein, with ethylene receptor AtERS1 or ER-bound *Arabidopsis* receptor Reticulon-like protein B2 (AtBTI2, Nziengui et al., 2007) was observed indicative of a proper localization of AtERS2 at the ER membrane in the *N. benthamiana* system. In subsequent *in planta* interaction studies, we analyzed fluorescence lifetime and anisotropy of a type-II receptor construct (AtERS2-mVenus) in combination with the type-I receptor isoform AtETR1. While changes in fluorescence lifetime refer to a direct interaction of different fluorophores (hetero-FRET), changes in anisotropy provide additional information about FRET processes between identical fluorophores (homo-FRET). Here, direct excitation of a fluorophore (mVenus) by another identical fluorophore in close proximity leads to a decreased anisotropy due to a depolarization effect. Hence, by analyzing fluorescence lifetime and anisotropy the homo- and hetero-FRET state of a donor fluorophore can be discriminated and thereby homo- and heteromeric interaction of the tagged protein (Gauthier et al., 2001; Bader et al., 2011). The results obtained in our FLIM experiments clarify that type-II receptor AtERS2 forms uniform homomeric complexes, but also heteromeric complexes with type-I receptor AtERS1 *in planta*. Thereby, the *in planta* interaction studies confirm our *in vitro* analysis which also attests homomeric complex formation of AtETR1 and AtETR2 but also tight heteromeric interaction of both receptor subfamilies. Previous studies proposed that the additional N-terminal α-helix in type-II receptors might serve as signal sequence (Chen et al., 2007). The tight affinity of type-II receptor homomeric and heteromeric complexes observed in our *in vitro* binding studies now suggests that the additional helix of type-II receptor may serve an increased complex stability. In principle, the tight affinity of type-II receptor complexes could also be related to sequence variation in individual helix segments. However, sequence alignment of both subtypes (Supplementary Figure S4) reveals that transmembrane helices of AtETR1 and AtETR2 are highly conserved (66% identical residues). In total, only five residues (G59/I88, A67/M96, L73/G102, A90/F119 and M104/T133 with the ETR1 residue and position given first) show low conservation. But even these residues have not experienced drastic changes in terms of physicochemical properties. Moreover, in a regular α-helix they do not align on the same surface. Consequently, a single of these minor changes then would have to account for the observed large differences in complex stability which is highly unlikely.

Heretofore, interactions of the ethylene receptor family were mainly attributed to the receptor GAF-domain (Xie et al., 2006; Grefen et al., 2008). However, we should bear in mind that the TM domain was not expressed in these studies. The quantitative binding studies presented in this work demonstrate for the first time that the isolated TM domain substantially contributes to receptor complex formation and probably plays a major role in mediating receptor-receptor interactions. Furthermore, previous studies demonstrated that receptors are stabilized by covalent cysteine crosslinks, although their impact on complex formation was not addressed (Schaller et al., 1995; Schaller and Bleecker, 1995). To address this point, we mutated the two cysteines near the amino terminus of AtETR1 (positions 4 and 6) to serine in order to prevent disulfide bond formation in the TM domain of the receptor. The tight and highly specific binding of the purified ETR1-TM^C4SC6S^ mutant to either AtETR1 or AtETR2 together with previous genetic studies of Xie et al. (2006) suggest that disulfide linkage is not essential for AtETR1 signaling.

In summary, our studies demonstrate that the type-II receptor AtETR2 binds AtEIN2 and AtCTR1 with similar affinities as type-I receptor AtETR1 indicating a similar role of both receptor subfamilies in PPI-based signal transfer, although they may still differ in other signal output mechanism mediated by post-translational modification such as phosphorylation or ubiquitination (Moussatche and Klee, 2004; O’Malley et al., 2005; Chen et al., 2007; Kevany et al., 2007). Specifically, the similar affinity of both subfamilies for their downstream targets may explain functional redundancy of the ethylene receptor family, i.e., the common function of all members to repress the constitutive ethylene response, although individual receptors can adopt specific functions that are not replaceable by other isoforms (Liu and Wen, 2012). Moreover, the similar affinity of both subfamilies for CTR1 and EIN2 may indicate that both target the same site in the receptors. However, a sequence alignment of the CTR1 N-terminus and the EIN2 C-terminus, which have been identified in previous studies to mediate binding to the receptors, reveals no particular sequence element to support this idea. Still, we have to bear in mind that, in the end, the 3D structure of a protein determines the interaction. Hence, structures of receptor complexes with CTR1 and/or EIN2 will ultimately unravel the binding site and signal transfer mechanism for both downstream signaling proteins.

Noteworthy, both receptor isoforms efficiently and specially bind to representatives of each subfamily, although interaction with type-II receptor protomers is slightly preferred probably due to a stabilizing effect of the additional TM helix in these receptors. These mixed dimers or higher molecular weight oligomers may reflect functional synergism of the different receptor subtypes to control scope, scale, and pace of ethylene responses. The preferred association of ETR1 with type-II receptors determined in our *in vitro* binding studies is fully in line with previous co-purification experiments of ETR1 with tagged versions of ERS1, ETR2, ERS2, and EIN4 from *Arabidopsis* membrane extracts (Gao et al., 2008) underscoring the biological significance of quantitative studies on purified individual components of the signaling pathway. To further dissect the functional role of the receptor heteromeric complexes for ethylene signaling, *in planta* studies on chimeric receptors consisting of different subdomains from the two subfamilies in different ethylene *loss-of-function* backgrounds may prove promising.

## Data Availability

The datasets generated for this study are available on request to the corresponding author.

## Author Contributions

GG conceived the project, planned, designed, and supervised the research. MB and NB planned and performed experiments with the assistance of SW-P, SH, and RCB. AM assisted with statistical analysis. MB, GG, NB, AM, SW-P, and SH analyzed data. MB, NB, and GG wrote the manuscript. SW-P and AM critical edited the manuscript. SH, SW-P, BS, YS, and RS provided expertise and feedback.

### Conflict of Interest Statement

The authors declare that the research was conducted in the absence of any commercial or financial relationships that could be construed as a potential conflict of interest.

## Supplementary Material

The Supplementary Material file for this article can be found online at: https://www.frontiersin.org/articles/10.3389/fpls.2019.00726/full#supplementary-material
