# Supplementary material for: Molecular Analysis of Protein-Protein Interactions in the Ethylene Pathway in the Different Ethylene Receptor Subfamilies
Source: Front Plant Sci. 2019 Jun 7;10:726. doi: 10.3389/fpls.2019.00726 (PMC6566107; doi:10.3389/fpls.2019.00726)
Supplement: Supplementary file 1 [file Data_Sheet_1.docx]

Supplementary Material

**Supplementary Table S1:** Primer list for molecular cloning

| **Oligonucleotide name** | **Oligonucleotide sequence** |
| --- | --- |
| pGEX-4T-1-TEV-ETR2-F | 5´-[Phos]GAAAATCTGTATTTTCAGGGCCCGGGTGTGAAAGAAATC-3´ |
| pGEX-4T-1-TEV-ETR2-R | 5´-[Phos]ATCCGATTTTGGAGG-3´ |
| pGEX-4T-1-TEV-ETR2-H_10_-F1 | 5´-[Phos]CATCATCATCATCACTGACTGACGATCTGCCTCG-3´ |
| pGEX-4T-1-TEV-ETR2-H_10_-F2 | 5´-[Phos]GCATCATCATCATCATCATCATCATCATCACTGACTGACG-3 |
| pGEX-4T-1-TEV-ETR2-H_10_-R | 5´-[Phos]AGCAGCTGATCGGCCTG-3´ |
| TOPO-ERS1-F | 5´-CACCATGGAGTCATGCGATTG-3´ |
| TOPO-ERS1-R | 5´-CCAGTTCCACGGTCTG-3´ |
| TOPO-ERS2-F | 5´-CACCATGTTAAAGACATTGTTAGTCC-3´ |
| TOPO-ERS2-R | 5´-GTGGCTAGTAGACGGAGGAGTTG-3´ |
| pABindmVmC-ERS2-F1 | 5´-ATGGTGAGCAAGGGC-3´ |
| pABindmVmC-ERS2-R1 | 5´-CGCTGTAGGTATCTCAGTTCGGTGTAGGTCGTTCGCTCC-3´ |
| pABindmVmC-ERS2-F2 | 5´-GGAGCGAACGACCTACACCGAACTGAGATACCTACAGCG-3´ |
| pABindmVmC-ERS2-R2 | 5´-GTGTTTGACAGGATATATTGGCGGGTAAAC-3´ |
| pABindmVmC-ERS2-F3 | 5´-GTTTACCCGCCAATATATCCTGTCAAACAC-3´ |
| pABindmVmC-ERS2-R3 | 5´-TGTACAAACTTGTTGATAGCTTGGCG-3´ |
| pABindmVmC-ERS2-F4 | 5´-CAACAAGTTTGTACAAAAAAGCAGGCTCCGC-3´ |
| pABindmVmC-ERS2-R4 | 5´-CCTCGCCCTTGCTCACCATCTTGTACAGCTCGTCCATGC-3´ |


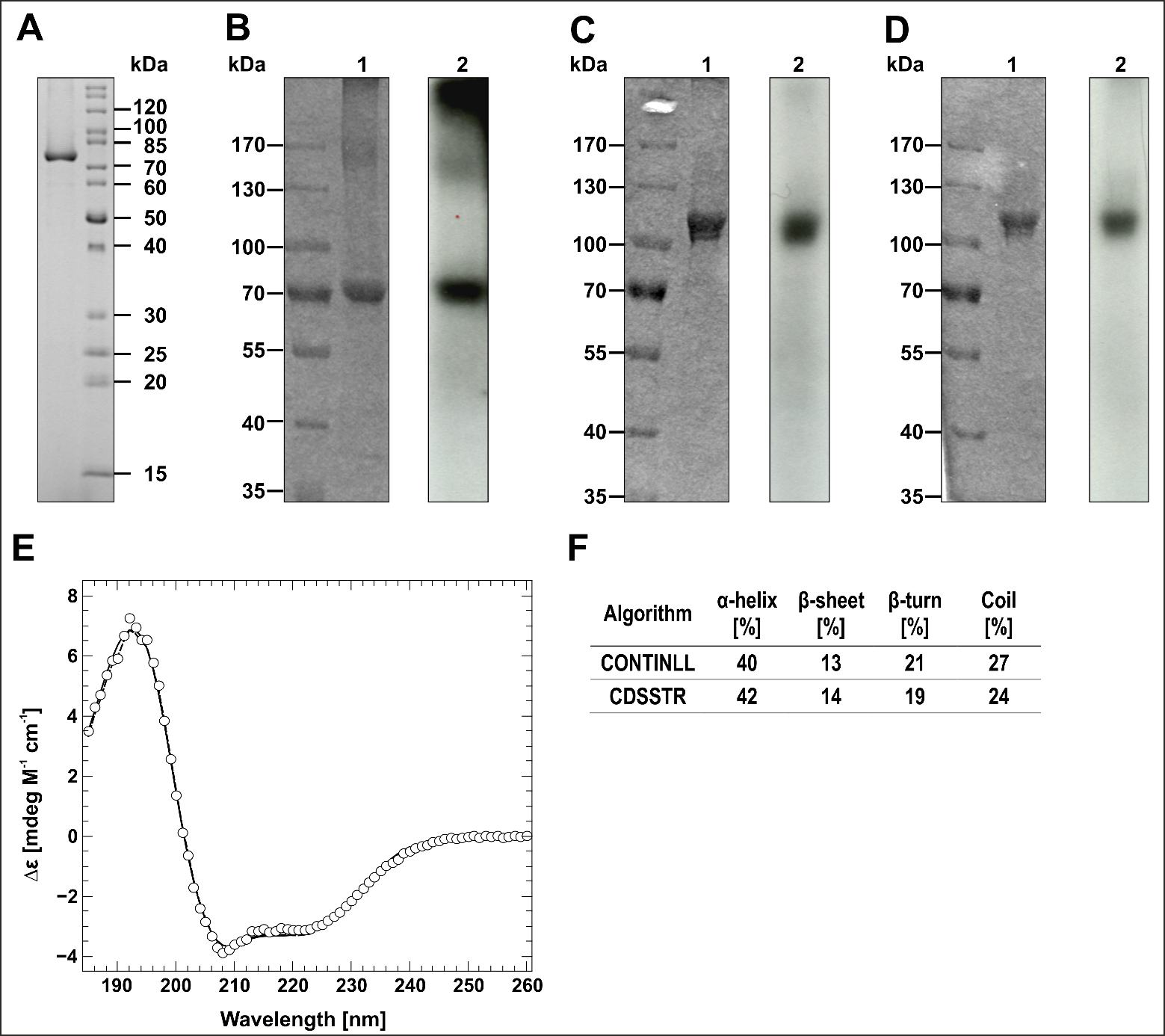


**Supplementary Figure S1:** (A) Recombinant receptor AtETR1 was heterologously expressed *in E. coli* strain C43 (DE) and purified by IMAC. Autokinase activity of purified AtETR1 (B), AtETR2 in the presence of magnesium (C) and AtETR2 in the presence of manganese as cofactor (D). Autoradiography was detected for six-days. Samples were analyzed by Comassie stained SDS gel (1) and autoradiography (2). (E) Far-UV spectra of purified AtETR1 was collected from 10 spectra measured with 1 nm bandwidth and a scanning speed of 50 nm/min. (F) Secondary structure content of purified AtETR1 from (E), calculated with CONTINLL (solid line) and CDSSTR (dashed line).

**
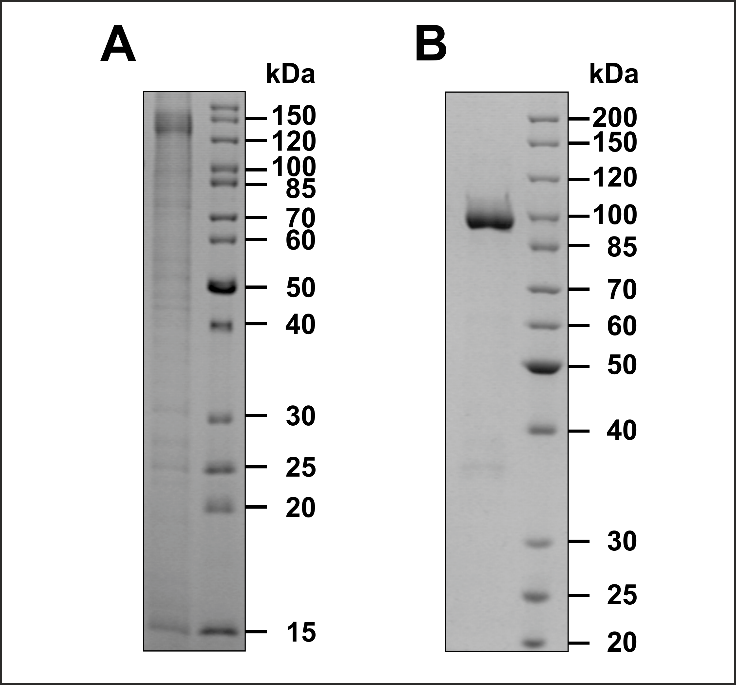
**

**Supplementary Figure S2:** Purification of His-tagged AtCTR1-CFP (A) and EIN2^479-1294^ (B) by IMAC. Purified proteins were analyzed by Coomassie stained SDS gel.


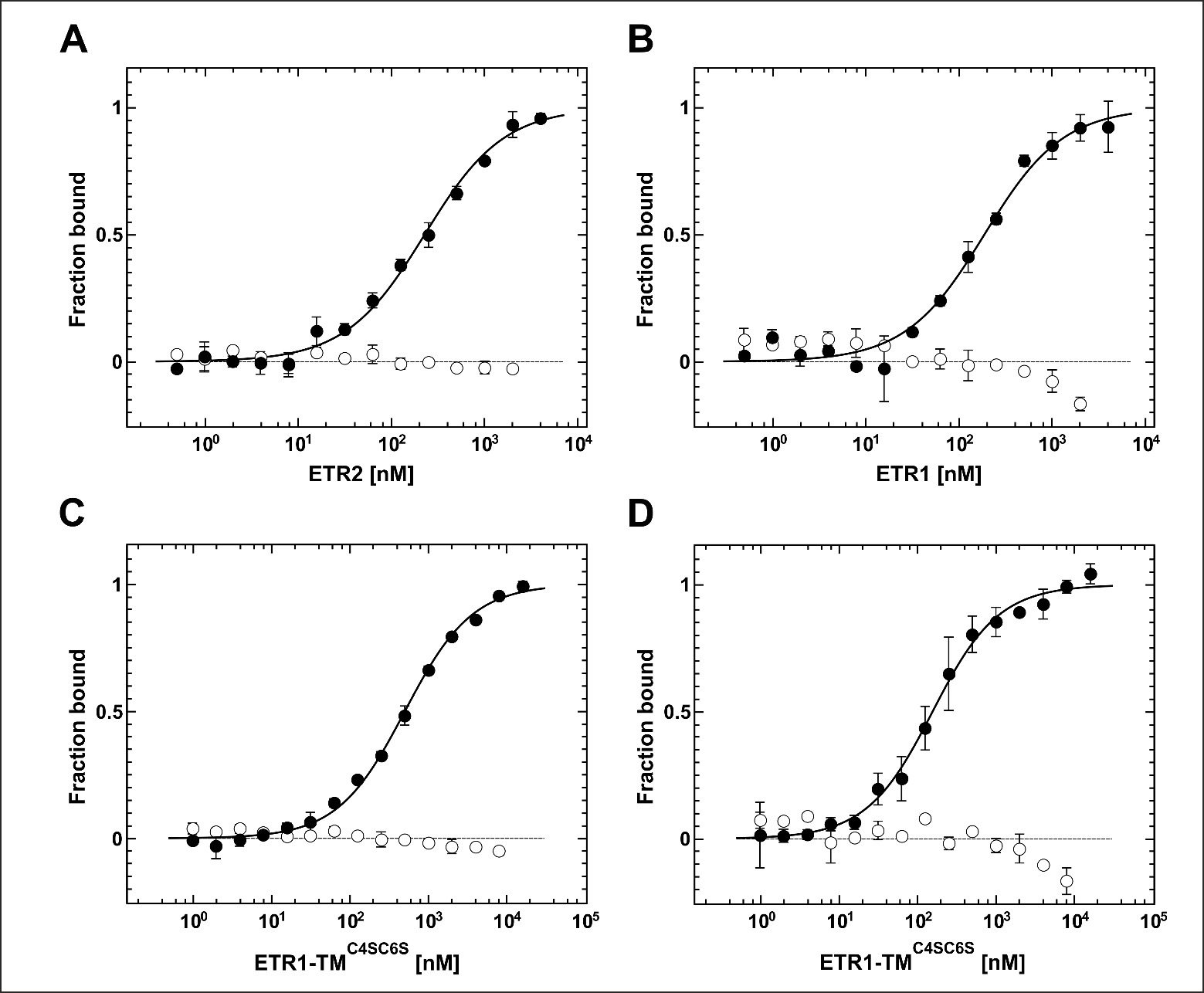


**Supplementary Figure S3:** MST-studies of receptor complex formations. (A) Determination of K_d_ value of AtETR2 binding to labelled AtETR1 (●) based on MST is illustrated. A K_d_ value of 177(18) nM was calculated. (B) For AtETR2 – AtETR1 (●) complex formation a K_d_ value of 217(14) nM was obtained. (C) Interaction binding studies of the isolated AtETR1 transmembrane domain (AtETR1-TM^C4SC6S^) to labelled AtETR1 (●) revealed a K_d_ value of 491(20) nM. (D) Titration of AtETR1-TM^C4SC6S^ to labelled AtETR2 (●) resulted in a K_d_ of 145(9) nM. Chemically denatured receptors (○) indicate no further binding event with AtETR1 (A, C) and AtETR2 (B, D) respectively. All data represent the mean(SD) of three independent triplicates in receptor – receptor binding studies (●) or duplicates in control measurements (○).

**Supplementary Table S2:** FLIM measurement data, related to Figure 8A

| **Sample** | **No.** | **Exponential fit** | **n** | **mean τ [ns]** | **SD** | **E_FRET_ [%]** | ***t-test*** | **p-value** |
| --- | --- | --- | --- | --- | --- | --- | --- | --- |
| **Free mVenus** | 1 | Mono-exp. | 34 | 2.98 | 0.05 |  |  |  |
| **ERS2-mV** | 2 | Mono-exp. | 113 | 2.90 | 0.06 |  |  |  |
| **ERS2-mV  + ERS1-mC** | 3 | Double-exp. | 68 | 2.56 | 0.10 | 11.7 | 2; 3 | < 0.0001 |
| **ERS2-mV  + ERS2-mC** | 4 | Double-exp. | 18 | 2.59 | 0.11 | 10.6 | 2; 4 | < 0.0001 |
| **ERS2-mVmC** | 5 | Double-exp. | 50 | 2.29 | 0.09 | 20.8 | 2; 5 | < 0.0001 |
| **ERS2-mV  + BTI2-mC** | 6 | Double-exp. | 62 | 2.90 | 0.08 | 0.0 | 2; 6* | 0.7422 |

mVenus fluorescence lifetime was measured by confocal microscopy in transiently expressing *N. benthamiana* leaf epidermal cells (co)infiltrated with the appropriate sample. A double or mono-exponential fitting was used. n = number of analyzed images, τ = mean mVenus fluorescence lifetime, SD = standard deviation, E_FRET_ = FRET efficiency of the respective FRET pair (No. 3, 4, 5, 6) with regard to the donor-only sample (No. 2), *t-test* = Data used for statistical analysis by Welch’s t-test and Mann-Whitney test (*) to determine the p-value.

**Supplementary Table S3:** Fluorescence anisotropy *r* measurement data, related to Figure 8B

| **Sample** | **No.** | **n** | **mean *r*** | **SD** | ***t-test*** | **p-value** |
| --- | --- | --- | --- | --- | --- | --- |
| **Free mVenus** | 1 | 34 | 0.298 | 0.012 |  | - |
| **ERS2-mV** | 2 | 113 | 0.273 | 0.015 | 1; 2 | < 0.0001 |
| **ERS2-mV  + ERS1-mC** | 3 | 68 | 0.294 | 0.017 | 2; 3  1; 3 | < 0.0001  0.2119 |
| **ERS2-mV  + ERS2-mC** | 4 | 18 | 0.318 | 0.010 | 2; 4 | < 0.0001 |
| **ERS2-mVmC** | 5 | 50 | 0.310 | 0.012 | 2; 5 | < 0.0001 |
| **ERS2-mV  + BTI2-mC** | 6 | 62 | 0.275 | 0.015 | 2; 6 | 0.4007 |

mVenus anisotropy *r* was measured in transiently expressing *N. benthamiana* leaf epidermal cells (co)infiltrated with the appropriate sample. n = number of analyzed images, *r* = mean anisotropy of mVenus, SD = standard deviation, *t* = Data used for statistical analysis by Welch’s t-test to determine the p-value.


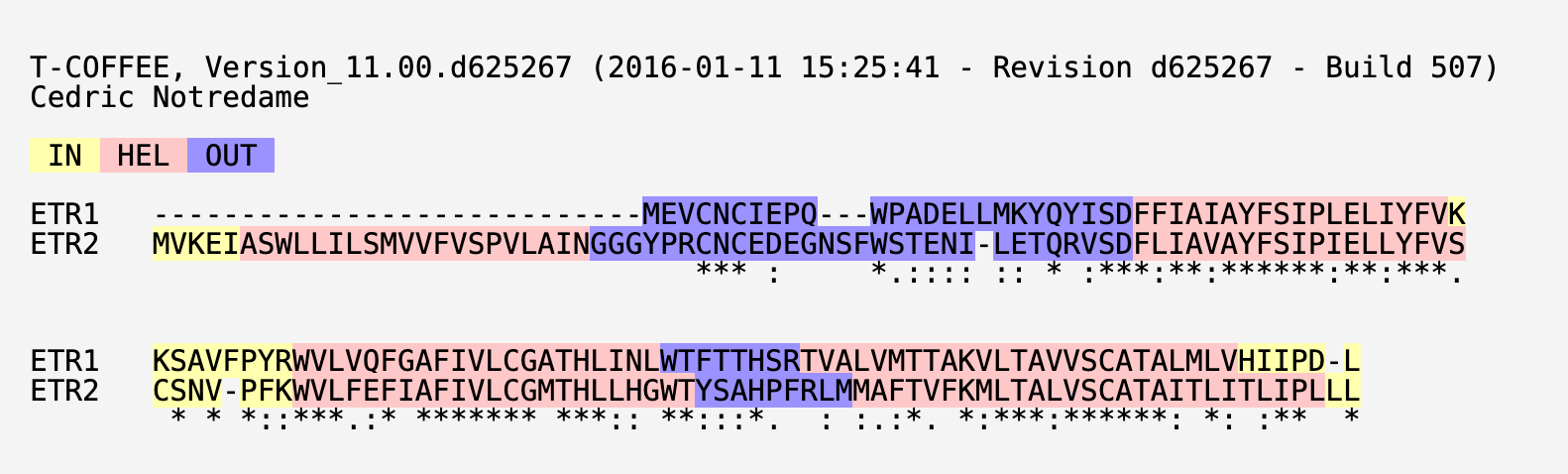


**Supplementary Figure S4: Sequence alignment of transmembrane regions of *Arabidopsis* ethylene receptors ETR1 and ETR2.** Asterisk (*) indicates positions which have a single, fully conserved residue. Colon (:) indicates conservation between groups of strongly similar properties - scoring > 0.5 in the Gonnet PAM 250 matrix. Period (.) indicates conservation between groups of weakly similar properties - scoring =< 0.5 in the Gonnet PAM 250 matrix. Alignment was produced by PSI/TM-Coffee (Chang et al., 2012).

**References**

Chang, J. M., Di Tommaso, P., Taly, J. F., and Notredame, C. (2012). Accurate multiple sequence alignment of transmembrane proteins with PSI-Coffee. *BMC Bioinformatics* 13, 1–7. doi:10.1186/1471-2105-13-S4-S1.
